# Physiology of Red Cell Lineage: From Erythroblast Progenitors to Mature Red Blood Cell

**DOI:** 10.3390/ijms24119715

**Published:** 2023-06-03

**Authors:** Sarah Ducamp, Mariano A. Ostuni

**Affiliations:** 1 Department of Pathology, Boston Children’s Hospital, Boston, MA 02115, USA; sarah.ducamp@childrens.harvard.edu; 2 Université Paris Cité and Université des Antilles, INSERM U1134, BIGR, F-75014 Paris, France

Red blood cells (RBC) are the most abundant cells in mammals. Their primary function is transporting and exchanging O_2_ and CO_2_ between the circulation and the organs. Every second, an adult human bone marrow produces around two million reticulocytes, released to the circulation to become mature RBC. Erythropoiesis is a tightly regulated differentiation process producing RBC from hematopoietic stem cells. It requires fine-tuning and coordinated regulation of specific gene expression leading to specific erythroid programs such as the massive heme and globin synthesis. It also needs support from non-erythroid cells, such as nursing macrophages of the erythroid island, and some very peculiar cell modifications, such as enucleation or organelle clearance, which give RBC their unique deformability in the bloodstream [1,2,3,4]. Decreases in hemoglobin content, lower RBC numbers, or changes in RBC properties or lifespan are associated with several types of anemia that are public health challenges.

For this Special Issue, we have gathered ten articles exploring different research topics on non-malignant hematology, including both basic and applied research. These studies investigate mechanisms involved in erythroid physiology and pathology. As Guest Editors, we aimed to balance new results with thorough reviews. Five teams presented novel findings contributing to our understanding of erythropoiesis [5,6,7,8,9]. Six teams critically evaluated our current knowledge and defined novel innovative questions for future research [7,10,11,12,13,14]. 

One of the strengths of this Special Issue is the broad range of topics covered. The articles in this Special Issue provide a comprehensive overview of the molecular mechanisms that regulate erythropoiesis [6,7,10,14], the red cell physiology [8,9], and the interaction between erythroblast and mature RBC with pathogens [9,10]. Different aspects of RBC diseases [10,12,13] and RBC-related diseases [5,11] are analyzed in this Special Issue. The growing evidence of the crucial interaction between RBC and cells from the red cell environment, such as macrophages [5,13] and endothelial cells [11], is noteworthy. 

**A journey from the nucleus of erythroblasts to the membrane of RBC.**

Vong and collaborators explore the role of acetylation, a post-translational modification, in erythroid differentiation. They focus on the role of the histone deacetylases (HDACs) protein family, which regulates several master genes during hematopoiesis. Interestingly, this review explores the lesser known function of cytosolic HDACs on early erythropoiesis. 

The review proposed by Medlock and Dailey presents the recently identified alternative pathways that enable a massive heme synthesis in erythropoiesis without the depletion key cellular metabolites. The authors also explore post-translational modifications regulating individual enzymes of the heme-synthesis pathways. These mechanisms could provide new targets for treating anemias and porphyrias [7]. 

Kumari and collaborators offer an original article exploring the role of TMCC2 in erythropoiesis. The physiological function of this endoplasmic reticulum transmembrane protein remains unknown, but its expression increases during terminal erythropoiesis. The authors generated a Tmcc2^−/−^ mice model that presents phenotypic characteristics shared with congenital dyserythropoietic anemias, generating new hypotheses regarding endoplasmic reticulum stress and erythroid maturation [6].

Rougé and collaborators explore the interaction between stomatin, a membrane protein proposed to regulate mechanosensitive channels, with pannexin 1, a red blood cell membrane protein able to drive ATP release from erythrocytes under stress conditions. They propose that stomatin plays a significant role in opening the PANX1 pore by being involved in a caspase-independent lifting of autoinhibition [8].

**From genetic disease to infections targeting erythroblasts and mature RBC.**

Maceckova and collaborators propose an extensive overview exploring why glucocorticoids are effective therapeutic agents for Diamond–Blackfan anemia (DBA). DBA is a rare genetic erythroid aplasia caused by defects in ribosomal proteins. The authors summarize the effect of glucocorticoids on normal erythropoiesis and the impact of these nonspecific agents in DBA-related pathways [12].

Laurance and collaborators review existing data concerning the role of RBC on central retinal vein occlusion (CRVO) pathophysiology. Even if CRVO has not been considered an RBC disease, growing evidence supports the concept of a role played by erythroid lineage in CRVO pathophysiology, especially related to occlusion events. This review also presents emerging data suggesting that disturbance of the RBC function could impact the vascular system [11].

Dumarchey and collaborators focus on a less explored aspect of malaria, a severe disease that affects many worldwide. Indeed, the development of anemia by patients infected with *P. falciparum* has been poorly investigated. The authors propose an overview of clinical and experimental studies investigating ineffective erythropoiesis in malaria patients but also in animal and in vitro models of malaria [10].

Other than intracellular infections, RBC are impacted by toxins derived from several pathogens such as *E. coli* hemolytic strains. Saffioti and collaborators characterize the mechanism involved in water transport induced by α-Hemolysin (HlyA) secreted by uropathogenic strains, and analyze how toxin-induced swelling and hemolysis might be coupled. These results provide new insights for understanding the mechanisms involved in HlyA signaling and cytotoxicity [9].

**RBC and macrophages: perturbed crosstalk with an old-time partner.**

Sesti-Costa and collaborators explore the mechanisms inducing stress erythropoiesis in sickle cell disease (SCD), a common but severe blood disorder. They focus on how stress erythropoiesis is potentially shifting the phenotype of nursing macrophages of the erythroid island to an inflammatory profile instead of its proliferation- and differentiation-inducing role [13]. 

These immune-phagocytic specialized cells are particularly affected in Gaucher disease (GD). Patients present with sphingolipid-overloaded macrophages. Dupuy and collaborators investigate erythrophagocytosis using RBC and macrophages derived from GD patients and healthy donors. Erythrophagocytosis of GD RBC is enhanced and induces the expression of antigen-presenting molecules CD1d and MHC-II, which could contribute to the pathophysiology of this disease [5].

**Conclusions**. Overall, the ten articles in this Special Issue provide valuable insights into the complex processes that govern RBC development and function in health and disease. They highlight the potential for future advances in this area of research. As of May 2023, each article has been viewed 700 to 2500 times, confirming the research community’s interest in RBC biology.
